# Allyl Isothiocyanate Exhibits No Anticancer Activity in MDA-MB-231 Breast Cancer Cells

**DOI:** 10.3390/ijms19010145

**Published:** 2018-01-04

**Authors:** Md. Abu Sayeed, Massimo Bracci, Veronica Ciarapica, Marco Malavolta, Mauro Provinciali, Ernesta Pieragostini, Simona Gaetani, Federica Monaco, Guendalina Lucarini, Venerando Rapisarda, Roberto Di Primio, Lory Santarelli

**Affiliations:** 1 Department of Clinical and Molecular Sciences, Polytechnic University of Marche, 60126 Ancona, Italy; sayeed.ru@gmail.com (M.A.S.); veronica.ciarapica@virgilio.it (V.C.); e.pieragostini@univpm.it (E.P); simonagae87@alice.it (S.G.); monaco_federica@virgilio.it (F.M.); guendalina.lucarini@univpm.it (G.L.); r.diprimio@univpm.it (R.D.P.); l.santarelli@univpm.it (L.S.); 2 Advanced Technology Center for Aging Research, Scientific and Technological Pole, Italian National Institute of Health and Science on Aging (INRCA), 60120 Ancona, Italy; m.malavolta@inrca.it (M.M.); m.provinciali@inrca.it (M.P.); 3 Occupational Medicine, Department of Clinical and Experimental Medicine, University of Catania, Via Santa Sofia 78, 95123 Catania, Italy; vrapisarda@unict.it

**Keywords:** breast cancer, allyl isothiocyanate, proliferation, apoptosis, cell cycle

## Abstract

It was reported recently that allyl isothiocyanate (AITC) could inhibit various types of cancer cell growth. In the present study, we further investigated whether AITC could inhibit the growth of human breast cancer cells. Unexpectedly, we found that AITC did not inhibit, rather slightly promoted, the proliferation of MDA-MB-231 breast cancer cells, although it did have inhibitory effect on MCF-7 breast cancer cells. Cytofluorimetric analysis revealed that AITC (10 µM) did not induce apoptosis and cell cycle arrest in MDA-MB-231 cells. In addition, AITC significantly (*p* < 0.05) increased the expression of *BCL-2* and *mTOR* genes and Beclin-1 protein in MDA-MB-231 cells. No significant changes in expression of *PRKAA1* and *PER2* genes, Caspase-8, Caspase-9, PARP, p-mTOR, and NF-κB p65 proteins were observed in these AITC-treated cells. Importantly, AITC displayed cytotoxic effect on MCF-10A human breast epithelial cell line. These observations suggest that AITC may not have inhibitory activity in MDA-MB-231 breast cancer cells. This in vitro study warrants more preclinical and clinical studies on the beneficial and harmful effects of AITC in healthy and cancer cells.

## 1. Introduction

Breast cancer has been a leading cause of cancer-associated death in females worldwide. Currently available treatments of this cancer show unsatisfactory efficacy. In addition to development of drug-resistance and side-effects of therapy, the risk of cancer recurrence after therapy is high. To find safe and effective drugs, research has focused on natural compounds because of their safe nature. Several compounds have been proposed as anti-breast cancer agent based on in vitro and animal studies [1,2].

Allyl isothiocyanate (AITC; 3-isothiocyanato-1-propene, CH_2_CHCH_2_NCS) is a sulfur containing organic compound and is an enzymatic hydrolysis product of the glucosinolate sinigrin present in various cruciferous vegetables, such as Brussels sprouts (8.9 μmol/g dry wt), cauliflower (9.3 μmol/g dry wt), cabbage (7.8 μmol/g dry wt), horseradish (0.91–144 μmol/g dry wt), kale (10.4 μmol/g dry wt), and wasabi (0.015–0.0162 μmol/g fresh wt) [3,4,5]. Its bioavailability is extremely high [6,7] and formulations for controlled release of AITC were developed recently [8,9]. Many studies have shown that AITC has various beneficial effects, including anti-inflammatory, neuroprotective, hepatoprotective, gastroprotective, anti-lipogenic/adipogenic, and antimicrobial effects [7,10,11,12,13,14,15]. Moreover, studies have shown that AITC displays anticancer activity. AITC inhibited proliferation through induction of apoptosis and cell cycle arrest in breast [16,17] and bladder [18,19] cancer cells as well as in brain malignant glioma [20], suppressed epidermal growth factor-stimulated invasion and migration in colorectal adenocarcinoma cells [21], inhibited proliferation via induction of apoptosis and cell cycle arrest in prostate cancer cells in vitro [22] and in vivo [23], and induced apoptosis in leukemia cells [24]. In addition to these studies, AITC has been reported to sensitize ovarian and lung cancer cells to chemotherapeutic drug cisplatin [25], and to exert synergistic therapeutic effects on lung cancer cells in combination with radiation [26].

Numerous molecules have been reported to play important roles in regulating apoptosis [27]. Caspase-8 and Caspase-9 are well known regulators of apoptosis. Activated Caspase-8 can activate Caspase-1, Caspase-3, Caspase-6, Caspase-7, and Bid [28,29]. Similarly, activated Caspase-9 can activate Caspase-3, Caspase-7. PARP (Poly (ADP-ribose) polymerase) is known to be involved in apoptosis [30] as well as in DNA repair system [31], and is one of the main targets of Caspase-3 [32]. Beclin-1 is important regulators of autophagy [33,34] and NF-κB (nuclear factor kappa-light-chain-enhancer of activated B cells) p65 is a member of NF-κB family which is important regulators of inflammation [35] andapoptosis [36]. p-mTOR (phospho-mammalian target of rapamycin) is a phosphorylated form of mTOR, which is involved in various cellular events including proliferation, autophagy [37]. It is well known that BCL-2 (B-cell lymphoma 2), mTOR, and PER2 regulate apoptosis, proliferation and autophagy, and circadian rhythms [38], respectively. PRKAA1 (5′-AMP-activated protein kinase catalytic subunit α-1) is the catalytic subunit of AMPK (5′ adenosine monophosphate-activated protein kinase), which is involved in regulating metabolic enzymes and as well inhibits mTOR through phosphorylation [39,40,41,42].

In the present study, we investigated whether AITC could inhibit the growth of breast cancer cells. Unexpectedly, we found that AITC did not inhibit, rather slightly promoted, the proliferation of MDA-MB-231 (negative for estrogen receptors, progesterone receptors, and human epidermal growth factor receptor 2) breast cancer cells. We also analyzed the level of Caspase-8, Caspase-9, PARP, Beclin-1, and p-mTOR proteins and the expression of *BCL-2*, *mTOR*, *PRKAA1*, and *PER2* genes in these cells after treatment with AITC and found that AITC did not affect the expression of some of these molecules. This finding suggests that the use of AITC for treating triple negative breast cancer may not be effective.

## 2. Results

### 2.1. AITC Did Not Inhibit MDA-MB-231 Cell Proliferation While Affected MCF-7 and MCF-10A Cells

**Figure 1:**
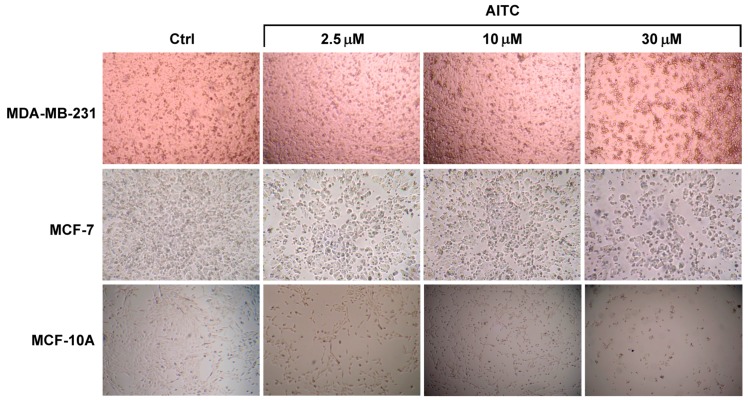
Representative photographs captured with 25× magnification of MDA-MB-231, MCF-7, and MCF-10A cells (control and after treatment with AITC for 48 h).

**Figure 2:**
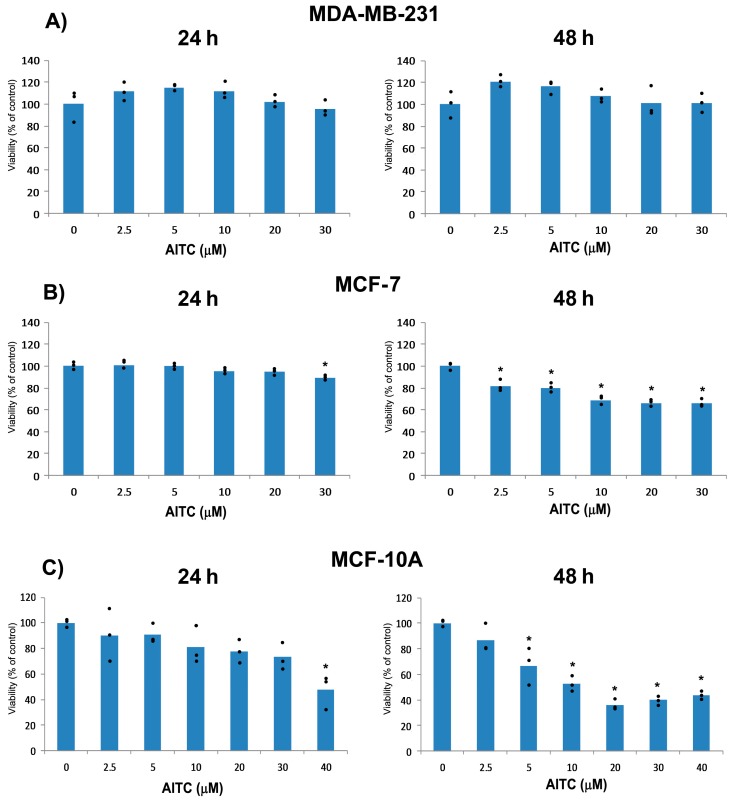
Effects of AITC on proliferation in MDA-MB-231, MCF-7, and MCF-10A cells. MDA-MB-231 (**A**); MCF-7 (**B**); and MCF-10A (**C**) cells were treated with various concentrations of AITC for 24 and 48 h, and then cell viability was determined by the MTT (methylthiazolyldiphenyl-tetrazolium bromide) assay. Values are presented as individual dots, and symbol asterisk indicates significant (*p* < 0.05) difference as compared to the control cells.

We planned the experiment to investigate whether AITC can inhibit proliferation of MDA-MB-231 breast cancer cells. For our study, we selected 2.5, 5, 10, 20, and 30 µM concentrations based on previous reports [16,26]. Cells were treated with various concentrations of AITC for 24 and 48 h. AITC did not inhibit, rather slightly increased, the proliferation of these cells (Figure 1 and Figure 2A). In contrast, AITC inhibited proliferation of MCF-7 cells in a dose and time-dependent manner (Figure 1 and Figure 2B). We also investigated the effect of AITC on cell viability of MCF-10A non-tumorigenic breast cells. MCF-10A cells were treated with AITC at 0, 2.5, 5, 10, 20, 30, and 40 µM for 24 and 48 h. Our results indicate that AITC shows toxic effects on this non-tumorigenic breast cell line (Figure 1 and Figure 2C). The IC_50_ values of AITC were 527.8 µM (at 24 h) and not calculable (at 48 h) for MDA-MB-231, 188.1 (at 24 h) and 126.0 µM (at 48 h) for MCF-7, 53.72 (at 24 h), and 14.23 µM (at 48 h) for MCF-10A.

### 2.2. AITC Did Not Induce Apoptosis and Cell Cycle Arrest

**Figure 3:**
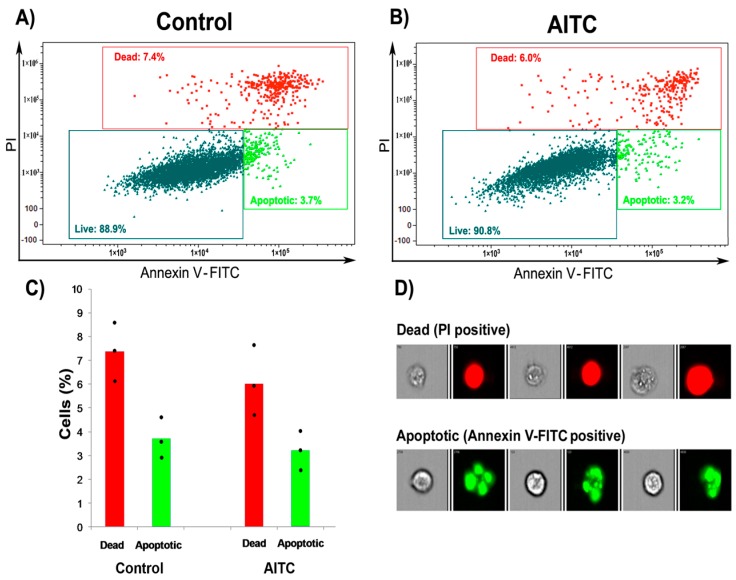
AITC did not induce apoptosis in MDA-MB-231 cells: (**A**,**B**) flow cytometric analysis of cell apoptosis; (**C**) histogram showing dead and apoptotic rates of control and AITC-treated cells; and (**D**) representative flow cytometric images of propidium iodide (PI; red fluorescence) and Annexin V-FITC (green fluorescence) positive cells.

Apoptosis was analyzed by flow cytometer in MDA-MB-231 cells after treatment with 10 µM AITC for 24 h. Approximately 3.2% and 6.0% of the AITC-treated cells were positive for Annexin V-FITC (Annexin V conjugated to green-fluorescent fluorescein isothiocyanate dye) and PI (propidium iodide) after 24 h, respectively (Figure 3B–D). In comparison, 3.7% and 7.4% of the control cells were positive for Annexin V-FITC and PI, respectively (Figure 3A,C,D). Our results indicate that AITC did not induce, rather slightly decreased, apoptosis in these cells.

**Figure 4:**
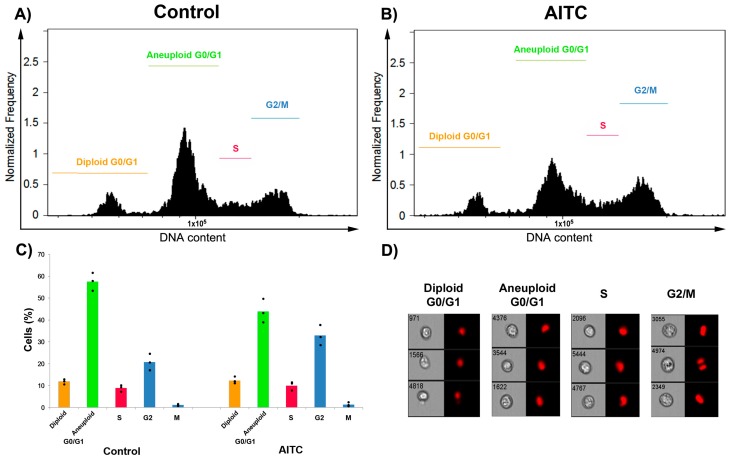
AITC did not induce cell cycle arrest in MDA-MB-231 cells: (**A**,**B**) flow cytometric analysis of cell cycle; (**C**) histogram showing rate of control and AITC-treated cells of different cell cycle phases; and (**D**) representative flow cytometric images of cell cycle phases.

Cell cycle control is important in cancer progression. Hence, we studied the effects of AITC on cell cycle progression in MDA-MB-231 cells. Cytofluorimetric analysis indicated that AITC did not induce the arrest of phases of the cell cycle significantly. Approximately 12.2%, 43.8%, 9.8%, 32.9%, and 1.2% of AITC-treated cells were noted in G_0_/G_1_ (diploid), G_0_/G_1_ (aneuploid), S, G_2_, and M phases, respectively (Figure 4B–D). By contrast, approximately 11.8%, 57.5%, 8.9%, 20.7%, and 1.1% of control cells were noted in G_0_/G_1_ (diploid), G_0_/G_1_ (aneuploid), S, G_2_, and M phases, respectively (Figure 4A,C,D). These results suggest that AITC has no ability to induce the cell cycle arrest in MDA-MB-231 cells.

### 2.3. AITC UpregulatedBCL-2 and mTOR Expression, While Induced No Changes in PRKAA1 and PER2 Expression

**Figure 5:**
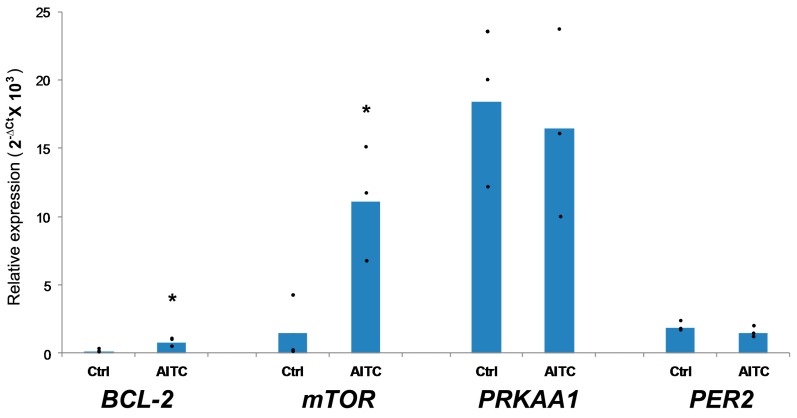
Relative gene expression levels of *BCL-2*, *mTOR*, *PRKAA1*, and *PER2* in MDA-MB-231 cells after treatment with 10 µM AITC for 48 h. * *p* < 0.05.

In this study, we also measured the expression levels of selected *BCL-2*, *mTOR*, *PRKAA1*, and *PER2* genes in MDA-MB-231 cells after treatment with AITC (10 µM) for 48 h. We observed that AITC significantly increased the expression of *BCL-2* and *mTOR* (Figure 5). In contrast, AITC did not affect the expression of *PRKAA1* and *PER2* (Figure 5). These results suggest that AITC may not inhibit, rather may promote, breast cancer cell growth.

### 2.4. AITC Did Not Affect Caspase-8, Caspase-9, PARP, p-mTOR, and NF-κB p65 Proteins Level, While Downregulated Beclin-1 Protein Level

**Figure 6:**
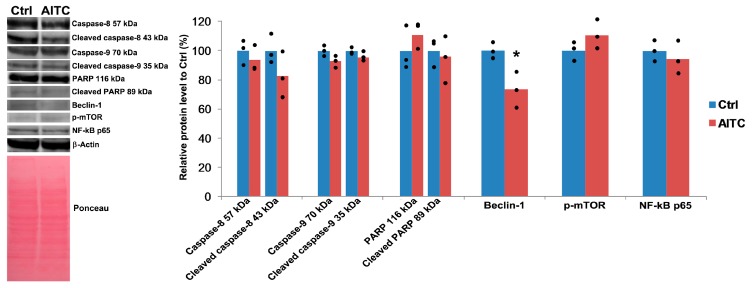
Protein levels of Beclin-1, Caspase-8, Caspase-9, PARP, p-mTOR, and NF-κB p65 in MDA-MB-231 cells after treatment with 10 µM AITC for 48 h. β-actin was used as a loading control. * *p* < 0.05.

Next, we performed experiments to study the expression levels of some selected Caspase-8, Caspase-9, PARP, Beclin-1, and p-mTOR proteins (Figure 6). These genes are aberrantly expressed in breast cancer. Several previous studies have reported that phytochemicals can modulate the expressions of these molecules in various cancer cells including breast cancer [1,43,44,45,46,47]. Hence, we analyzed the expression levels of these proteins by western blot technique. Cells were treated with 10 µM AITC for 48 h and we found that AITC did not affect the level of Caspase-8, Caspase-9, PARP, and p-mTOR, and NF-κB p65 significantly. In contrast, AITC decreased the level of Beclin-1 significantly. These results indicate that AITC shows no growth inhibitory effect on MDA-MB-231 cells.

## 3. Discussion

Many previous studies have shown that natural compounds can inhibit cancer initiation, development, and progression [48]. They have been proposed as potential cancer preventive agents, although some natural compounds such as isothiocyanates show carcinogenesis promoting effects [49].

In this study, we demonstrate that AITC cannot inhibit, rather can promote, the proliferation of MDA-MB-231 breast cancer cells. In contrast, AITC can inhibit the proliferation of MCF-7 breast cancer cells. Importantly, we observed that AITC significantly affected MCF-10A non-tumorigenic breast cells. We observed that AITC did not induce apoptosis and cell cycle arrest in MDA-MB-231 cells, as demonstrated by flow cytometry. We also found that AITC failed to alter the expression of Caspase-8, Caspase-9, PARP, and p-mTOR proteins, but reduced the expression of Beclin-1 proteins. In addition, AITC increased the expression of *BCL-2* and *mTOR* genes, and did not affect *PRKAA1* and *PER2* genes. We did not use the MCF-7 cells for our further study since our MTT results confirmed the previous report [17].

Apoptosis is an important biological process by which cell death occurs and is generally characterized by DNA and nuclear fragmentation, chromatin condensation, cytoplasmic membrane blebbing, and messenger RNA degradation. Apoptosis is regulated by various factors such as Fas receptors, Caspases, and BCL-2 family members [27,50,51]. This process is defective in various diseases including cancer. We found that AITC at 10 µM did not induce apoptosis in MDA-MB-231 breast cancer cells, as demonstrated by flow cytometry. Earlier studies have shown that AITC increased Caspase-8 in MDA-MB-231 cells [17] and HL60 human leukemia cells [24]; increased Caspase-9 in MDA-MB-231 [17] and MDA-MB-468 breast [16], UM-UC-3 bladder [19] cancer cells, and GBM 8401 brain malignant glioma cells [20]; decreased BCL-2 in MDA-MB-231 [17] and MDA-MB-468 [16] breast cancer cells, and PC-3 prostate cancer cells in vitro [22] and in vivo [23]; and increased PARP in MDA-MB-231 breast cancer [17] and SW620 colorectal adenocarcinoma cells [52]. We further studied the expression of these molecules in AITC-treated cells. Unexpectedly, we found that AITC (10 µM) did not affect the expression of Caspase-8, Caspase-9, and PARP proteins, as well as increased the expression of *BCL-2* gene. Our results suggest that AITC could not induce apoptosis in MDA-MB-231 cells.

Previous studies reported that AITC induced S phase cell cycle arrest in bladder cancer [53] and lung cancer [26] cells, and G_2_/M phase cell cycle arrest in bladder cancer [53], lung cancer [26], MDA-MB-468 breast cancer [16], brain malignant glioma [20], prostate cancer [22], and leukemia [24] cells. In this study, we observed that AITC did not induce cell cycle arrest significantly. These results suggest that AITC may not inhibit the growth of MDA-MB-231 cells.

Beclin-1 is aberrantly expressed in breast cancer and has been suggested as a therapeutic target [54]. We found that AITC decreased Beclin-1 level. Study reported that overexpressed Beclin-1 may promote autophagy in autophagy-defective breast cancer cells [55]. Thus, we suggest that AITC may promote disruption of autophagy in MDA-MB-231 cells.

The abnormal expression of mTOR is illustrated in breast cancer cells [56,57]. Previous studies reported that isothiocyanates can suppress mTOR in cancer cells [58,59,60,61]. In our study, we found that AITC upregulated *mTOR* expression, indicating AITC may promote breast cancer progression. Although *mTOR* was upregulated in AITC-treated cells, p-mTOR protein level was not significantly upregulated. We are currently unable to provide a definitive explanation for this paradox, but it is likely that this is not due to a simple difference in sensitivity between the two assays. Indeed, mTOR signaling pathway in MDA-MB-231 cells can be activated mainly via mTOR overexpression rather than phosphorylation of this molecule. A previous study confirms our assumption. This study reported that untreated MCF-7 cells show high p-mTOR levels and relatively low mTOR levels while MDA-MB-231 cells show an opposite profile [62].

NF-κB is overexpressed in breast cancer [63]. Various natural products including AITC have been shown to inhibit this oncogenic factor in breast cancer [46,64]. In this study, we observed that AITC did not affect NF-κB p65 significantly, which suggests that AITC has no ability to suppress this molecule in breast cancer.

It has been shown that activation of AMPKα1 and AMPKα suppressed the growth of colon cancer [65] and breast cancer [66] cells, respectively. Our result revealed that AITC did not affect the expression of *PRKAA1* in MDA-MB-231 cells, indicating AITC may not inhibit MDA-MB-231 breast cancer cell growth.

*PER2* is aberrantly expressed in breast cancer [67,68]. *PER2* may act as tumor suppressor gene [69,70]. Studies have shown that *PER2* can regulate cell cycle-related molecules Cyclin D and Cyclin E [71], and cell adhesion-related molecule β-catenin [72]. We found that AITC did not affect *PER2* expression, suggesting AITC may not modulate this molecule in MDA-MB-231 breast cancer cells.

Our results do not confirm previously published results about AITC on MDA-MB-231 cells [17], however changes in *mTOR* suggest a metabolic activation that can be addressed to the different medium used in the two studies. We used DMEM with high glucose, sodium pyruvate and l-glutamine, while the previous study used RPMI-1640 with l-glutamine [17]. DMEM high glucose is a medium with more nutrients compared to RPMI-1640 hence it can stimulate more the growth of MDA-MB231 cells. The increased metabolism might be responsible of the results obtained in this study.

The different response of AITC on proliferation of MDA-MB-231 compared to MCF-7 was further investigated. Since MDA-MB-231 cells do not have estrogen receptor (ER) and MCF-7 cells have, we hypothesized that the different responses of the two cell lines to AITC may be related to an interaction with ER. To test this hypothesis, we treated MCF-7 cells with ER inhibitor fulvestrant and AITC (Figure S1). Fulvestrant showed a synergic action with AITC at 24 h and an additive action at 48 h, suggesting that AITC may have an interaction with ER or estrogen signaling pathway.

In conclusion, the results of our present study indicate that AITC does not inhibit the proliferation of MDA-MB-231 breast cancer cells. AITC may not suppress, rather may promote, the aberrant expression of molecules related to proliferation, apoptosis, and autophagy in these breast cancer cells. Moreover, AITC can display cytotoxic effect on healthy breast cells. Although AITC has been suggested as a potential anticancer agent, this phytochemical may not have potential inhibitory activity in triple negative breast cancer cells. The results of this in vitro study warrant more preclinical and clinical studies on the beneficial and harmful effects of AITC in healthy and cancer cells.

## 4. Materials and Methods

### 4.1. Chemicals and Reagents

AITC, 3-(4,5-dimethylthiazol-2-yl)-2,5-diphenyltetrazolium bromide (MTT), propidium iodide (PI), ethanol, and isopropanol were purchased from Sigma-Aldrich (St. Louis, MO, USA). AITC was dissolved in ethanol at a concentration of 10 mM and stored at 4 °C. Dulbecco’s Modified Eagle Medium (DMEM), Dulbecco’s Modified Eagle Medium: Nutrient Mixture F-12 (DMEM/F12), and fetal bovine serum (FBS) were purchased from Euroclone (Pero, Italy). Annexin V-FITC apoptosis detection kit was purchased from eBioscience Inc. (San Diego, CA, USA), RIPA buffer was purchased from Thermo Fisher Scientific (Waltham, MA, USA). *BCL-2*, *mTOR*, *PRKAA1*, and *PER2* gene primers were purchased from Integrated DNA Technologies (Coralville, IA, USA). Primary antibodies against Caspase-8, Caspase-9, PARP, Beclin-1, p-mTOR, NF-κB p65, and β-actin, and secondary antibodies were purchased from Cell Signaling Technology (Danvers, MA, USA).

### 4.2. Cell Line and Culture Conditions

MDA-MB-231, MCF-7 human breast cancer cell lines and MCF-10A human breast epithelial cell line were purchased from Istituto Zooprofilattico Sperimentale della Lombardia e dell’Emilia Romagna (IZSLER, Brescia, Italy). MDA-MB-231 and MCF-7 cells were grown in DMEM with high glucose (4500 mg/L), sodium pyruvate (110 mg/L) and l-glutamine (584 mg/L) supplemented with 10%FBS and 1% penicillin–streptomycin. MCF-10A cells were grown in DMEM/F12 supplemented with 5% horse serum, 20 ng/mL EGF, 0.5 µg/mL hydrocortisone, 100 ng/mL cholera toxin, 10 µg/mL insulin and 1% penicillin–streptomycin. They were maintained at 37 °C and 5% CO_2_ in an incubator. The growth medium was changed after 48 or 72 h, and cells were trypsinized and subcultured when they reached 80–90% confluence.

### 4.3. Cell Viability Analysis

To analyze cell viability, we used MTT assay. Cells (2 × 10^4^/well) were seeded in 96-well plate. After 24 h, cells were treated with AITC at 0, 2.5, 5, 10, 20, 30, and 40 µM. After 24 h or 48 h of treatment, 10 µL of MTT (5 mg/mL in PBS) was added to each well and the plate was incubated again for 3 h at 37 °C in incubator. Next, medium was removed, 200 µL of isopropanol was added, and the absorbance was read at 595 nm [73]. Cell viability rate was calculated following the formula: cell viability (%) = (average OD value of AITC-treated cells/average OD value of control cells) × 100.

### 4.4. Apoptosis Analysis

For apoptosis analysis, MDA-MB-231 cells (1 × 10^5^/well) were cultured in 6-well for 24 h and treated with AITC at 10 µM. After 24 h of treatment, floating and adherent cells were collected and stained with Annexin V-FITC and PI following the manufacturer’s instructions. Stained cells were then analyzed using an imaging flow cytometer named Flow Sight (Amnis Corporation, Seattle, WA, USA) and IDEAS Software version 6.2 (EMD Millipore, Burlington, MA, USA).

### 4.5. Cell Cycle Analysis

MDA-MB-231 cells (3.3 × 10^5^) were seeded in T25 flasks for 24 h and treated with AITC at 10 µM for 24 h. After that both floating and adherent cells were collected and fixed in 70% ethanol (cold) for 24 h. Ethanol was removed, cells were then washed twice with PBS, and resuspended in 1 mL of PBS containing 0.02 mg/mL PI, 0.2 mg/mL DNase-free RNase A, and 0.1% Triton X-100, and incubated for 15 min at 37 °C in incubator. After incubation, cells were analyzed using a flow cytometer [74].

### 4.6. Western Blotting

MDA-MB-231 cells (3.3 × 10^5^) were seeded in T25 flasks. After 24 h, cells were treated with 10 µM AITC for 48 h, and collected for protein expression analysis. Protein extraction and quantification as well as western blotting were performed according to protocol described elsewhere [75,76,77].

### 4.7. RNA Extraction and Real-Time PCR

MDA-MB-231 cells (3.3 × 10^5^) were seeded in T25 flasks. After 24 h, cells were treated with 10 µM AITC for 48 h. After incubation, the cells were collected for RNA extraction. Total RNA was isolated using the RNeasy Mini Kit (QIAGEN, Hilden, Germany) according to the manufacturer’s protocol. RNA quality and quantification were evaluated with a Nanodrop 1000 spectrophotometer (Thermo Scientific, Wilmington, DE, USA). cDNA was synthesized using the High-Capacity cDNA Reverse Transcription Kit (Applied Biosystems, Foster City, CA, USA) according to the manufacturer’s protocol. Gene expression levels were assayed using the TaqMan system (Applied Biosystems, Foster City, CA, USA). GAPDH was used as a housekeeping gene. The *C*t (threshold cycle) value of each gene of interest was normalized to Ct value of GAPDH and the relative gene expression data were analyzed using the equation: 2^−Δ*C*t^.

### 4.8. Statistical Analysis

Data were collected from three independent experiments. The average of intra-assay replications is presented as individual dots on top of bar graphs whose height represent the average of all the experiments. One-way ANOVA with post-hoc Tukey HSD (in the case of multiple independent groups) and Student’s *t*-Test (in the case of two independent groups) with Bonferroni’s correction (in the case of multiple testing) were used to evaluate the statistical significance and statistical analyses were performed using SPSS 19.0 (Statistical Package for the Social Sciences Inc., Armonk, New York, NY, USA) software. In figures, asterisk indicates the significant differences of data in comparison with the control. Data were considered statistically significant at *p* < 0.05. IC_50_ values were calculated using GraphPad Prism 7.03 (GraphPad Software, San Diego, CA, USA).

## Supplementary Materials

Supplementary materials can be found at www.mdpi.com/1422-0067/19/1/145/s1.
