# Supplementary material for: Allyl Isothiocyanate Exhibits No Anticancer Activity in MDA-MB-231 Breast Cancer Cells
Source: Int J Mol Sci. 2018 Jan 4;19(1):145. doi: 10.3390/ijms19010145 (PMC5796094; doi:10.3390/ijms19010145)
Supplement: Supplementary file 1 [file ijms-19-00145-s001.pdf]

Supplementary Materials: Allyl Isothiocyanate Exhibits No Anticancer Activity in MDA-MB-231 Breast Cancer Cells

Md. Abu Sayeed, Massimo Bracci, Veronica Ciarapica, Marco Malavolta, Mauro Provinciali, Ernesta Pieragostini, Simona Gaetani, Federica Monaco, Guendalina Lucarini, Venerando Rapisarda, Roberto Di Primio and Lory Santarelli

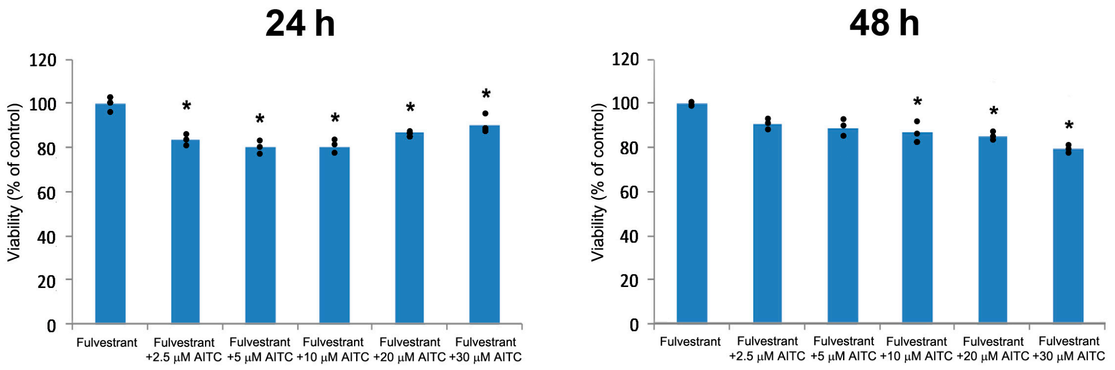

Figure S1. Effects of AITC on proliferation in MCF-7 cells treated with 100 nm Fluvestrant and various concentrations of AITC for 24 and 48 h, then cell viability was determined by the MTT assay. Values are presented as individual dots and symbol asterisk indicates significant ($p < 0.05$) difference as compared to the cells treated with Fluvestrant alone.
